# LED lighting (350-650nm) undermines human visual performance unless supplemented by wider spectra (400-1500nm+) like daylight

**DOI:** 10.1038/s41598-026-35389-6

**Published:** 2026-01-23

**Authors:** Edward M. Barrett, Glen Jeffery

**Affiliations:** 1 Institute for Environmental Design and Engineering, University College London, London, UK; 2 Institute of Ophthalmology, University College London, London, EC1V 9EL UK

**Keywords:** LED lighting, Metabolism, Infrared, Neuroscience, Environmental social sciences, Engineering

## Abstract

Life evolved under broad spectrum sunlight, from ultraviolet to infrared (300–2500 nm). This spectrally balanced light sculpted life’s physiology and metabolism. But modern lighting has recently become dominated by restricted spectrum light emitting diodes (350–650 nm LEDs). Absence of longer wavelengths in LEDs and their short wavelength dominance impacts physiology, undermining normal mitochondrial respiration that regulates metabolism, disease and ageing. Mitochondria are light sensitive. The 420–450 nm dominant in LEDs suppresses respiration while deep red/infrared (670–900 nm) increases respiration in aging and some diseases including in blood sugar regulation. Here we supplement LED light with broad spectrum lighting (400–1500 nm+) for 2 weeks and test colour contrast sensitivity. We show significant improvement in this metric that last for 2 months after the supplemental lighting is removed. Mitochondria communicate across the body with systemic impacts following regional light exposure. This likely involves shifting patterns of serum cytokine expression, raising the possibility of wider negative impacts of LEDs on human health particularly, in the elderly or in the clinical environment where individuals are debilitated. Changing the lighting in these environments could be a highly economic route to improved public health.

## Introduction

Ambient light impacts on human health. Sunlight, under which life evolved, extends over approximately 300–2500 nm. Older incandescent lighting common until recently has a similar spectral range. But because our visual sensitivity is limited to 400–700 nm we are unaware of infrared light (approximately 700–2500 nm). However, light in the built environment is now driven by light emitting diodes (LEDs), whose restricted spectrum (approximately 350–650 nm) is designed around our visual sensitivity and consequently is economic^1^.

Typical LED lighting produces strong elements in the shorter blue wavelengths (420–450 nm) with a second yellow peak which drops swiftly above 650 nm, with little light above 700 nm^1^. Short wavelength exposure in animals in the range of 420–450 nm reduces mitochondrial function, which provides the energy for physiological performance in the form of adenosine triphosphate (ATP). This short wavelength light reduces mitochondrial complex activity and ATP production, in a highly conserved manner. Hoh Kam et al. showed a significant decrease in mitochondrial enzymatic activity in fruit flies for complexes I-IV under 420 nm light^2^. Kaynezhad et al. used broadband near infrared spectroscopy (bNIRS) imaging the mouse retina and reported significant instability of deoxygenated haemoglobin and oxidised cytochrome-c-oxidase after exposure to 420 nm light. This instability remained significant through a 1 h recovery period when the light was withdrawn^3^. Short wavelength light (420 and 450 nm) also results in increased body weight. Hussaini et al. demonstrated that mice exposed to these wavelengths gaining weight rapidly compared to controls over the course of eight weeks^4^. Shorter wavelengths in similar ranges are also associated with reduced lifespan. Nash et al. revealed a 50% drop in the median lifespan of fruit flies exposed to unfiltered white LED light relative to those kept in darkness, but only 4% drop if this LED light was passed through a yellow filter, blocking the shorter wavelength light^5^. This negative influence is likely due to mitochondrial absorption by porphyrin that may increase proinflammatory oxygen singlet production reducing mitochondrial function as proposed by Kaynezhad et al.^3^.

Longer wavelengths (700 nm+) penetrate deeply and those in sunlight can be measured passing through the human body^6^. These are absent from standard LEDs but present in sunlight and incandescent lighting. Their presence increases mitochondrial performance and ATP production, particularly when challenged by age or disease. Gkotsi et al. demonstrated significantly increased ATP production in the retina, cortex, and thalamus of mice following exposure to 670 nm light^7^. Calaza et al. revealed a 50% increase in ATP in eight-month-old complement factor H knock out mice that have a mitochondrial deficit and are used as a murine model of macular degeneration^8^.

Increased mitochondrial performance is associated with increased lifespan and enhanced mobility. Begum et al. demonstrated using fruit flies that exposure to 670 nm resulted in a positive divergence of ageing survival rates of 10% at 4 weeks of age and up to nearly 180% by 8 weeks of age. The older animals also displayed an almost doubling of mobility against controls^9^. Neonicotinoid insecticides specifically target mitochondrial respiration inducing Parkinson like symptoms of immobility resulting in death. Here 670 exposure reversed damaged ATP levels to normal and corrects mobility and lifespan issues^10^.

Increased mitochondrial activity should result in reduced blood sugars and increased oxygen consumption as mitochondria use both in respiration. Powner and Jeffery found both in bumble bees exposed to 670 nm light^11^. The same authors translated this to humans showing again, reduced blood sugars and increased oxygen consumption in a standard glucose tolerance test following 15 min of 670 nm exposure. Here the spike in blood glucose was reduced significantly by around 27%^12^.

Changes in physiology produced by longer wavelengths translate to improved function. Shinhmar et al. revealed improved colour contrast sensitivity in humans after 3 min of morning exposure to 670 nm light^13^. Hence, exposure to different ends of the spectrum that impact differentially on mitochondria can translate into changes in key physiological metrics.

Similar changes are found at the population level. Those spending more time in sunlight generally have improved health including reduced incidents of cardiovascular disease and the incidence of cancer. They also have lower rates of type 2 diabetes^14,15^.

In this study we confront the impact of LED lighting on human visual performance by measuring colour contrast detection in an LED illuminated working environment that is then supplemented with incandescent lighting. The hypothesis is that LED lighting suppresses mitochondrial function in the retina and that this can be corrected by introduction of wide spectrum incandescent lights. The results highlight the potential damaging influence of LED lighting on human performance.

## Methods

The Subjects and their environment: The study was conducted in accordance with the Declaration of Helsinki and approved by University College London research ethics committee (16547/001). It was undertaken in University College London buildings in October to December. In October local daylight hours were approximately 10.37 with 75% cloud cover. In November local daylight hours were approximately 8.45 with 55% cloud cover. In December local daylight hours were approximately 7.5 with 90% cloud cover. Local sunset time in October is approximately 18.30. In November it is approximately 16.15 and in December it is approximately 16.00. Consequently, many subjects would be returning home after sunset in November and December. Subjects worked approximately 8 h a day 5 days a week and travel to and from work via public transport that was illuminated by LED devices. Most subjects did not leave the building in which they worked during the working day in these months. For those that did it was commonly for less than 15 min at lunch time. Within the work environment subjects were free to move around. Here the internal lighting they experienced was consistently LED based. Hence, natural daylight exposure during this latter part of the year was limited. We could not control for weekend exposure, however subjects homes were consistently illuminated with LEDs and because the weather in the UK at this time of year is inclement, their time outside buildings can be expected to be limited.

Each participant provided written informed consent prior to testing and data generated was anonymised. Subjects (*N* = 22) were of both sexes and between the ages of 23 and 65 years. Prior to the experiment all subjects were asked to confirm normal corrected visual function and general good systemic health. This was undertaken in a simple interview prior to their inclusion in the study. All were healthy without visual or other health problems. Experimental subjects (*N* = 11) worked exclusively under LED lighting in the back of the Here East building on the north side, > 50 m from what little light did manage to penetrate the entrance doors when open. The LED lighting delivered an illuminance of 1000 lx at working height, with a correlated colour temperature (CCT) of 4000 K, and a TM-30 average colour fidelity index, *R*_f_, of 91. The infrared light that was introduced was provided by tungsten desk lamps placed around the working space was non-uniform. The visible component of the 60 W tungsten bulbs was small when compared with the 1000 lx of LED. The test subjects were not expected to use these as task lamps. The LED lighting delivered an irradiance of 3.7 W/m2 on the horizontal working plane.

Control subjects (*N* = 11) worked in similar environments under LED lighting without direct sunlight. The LED lighting delivered an illuminance of 900 lx at working height, with a CCT of 3000 K and a *R*_f_ of 85. The colour contrast tests were performed in a darkened room where the only light came from the test itself. There were no requirements restricting other light exposure patterns during the study.

The experimental location: Subjects worked at UCL Here East, a media and innovation complex located in East London (London E15 2GW), originally built as a press and broadcast centre for the London 2012 Olympics and subsequently repurposed as a campus. UCL Here East occupies part of the Broadcast Centre, taking up the ground and first floor of unit B. The footprint of the building is deep, with daylight only able to enter through the glazing at the front of the building. This glazing uses an infrared blocking film, which can be revealed using infrared photography.

A Canon 500D digital camera was modified to replace the infrared blocking layer with clear glass that passes infrared wavelengths. This was used in conjunction with filters that block visible and infrared wavelengths to explore the presence and absence of infrared light. Spectral measurements were made with two spectrophotometers (Ocean Optics SR-6XR250-50 and FLAME-NIR) with optic fibre and cosine correctors used to collect the incandescent spectra in the shorter and longer wavelengths.

Incandescent desk lighting was introduced into the work environment using desk lamps with 60 W clear Edison bulbs (Polaris UK) placed on work benches. All subjects had worked in this LED-lit environment for more than 2 years. Desk lamps with incandescent bulbs were introduced onto the benches where experimental subjects spent the majority of their time. They were given the incandescent lighting for 2 weeks and, while they spend the majority of their time working near these lights, they were free to move around and leave their desks as they wished. The introduced light showed a high degree of reflectance from the work surfaces.

Colour contrast testing: All subjects were tested for colour contrast ability using ChromaTest prior to the introduction of incandescent lighting and then again 2 weeks later. This test must be carried out in a darkened room, so a nearby windowless room was set aside for this purpose. The incandescent lighting was then removed and subjects retested at 4 and 6 weeks. Hence, this element of the experiment was a before and after design which avoids between subject variability. However, there was also a separate control group (*N* = 11) composed of subjects that worked under LED lighting similar to those in the experimental group.

ChromaTests is a sensitive measure of colour contrast detection of letters presented in a random order against a noisy visual background in either tritan (blue) or protan (red) visual axes^13^. If subjects correctly identify a letter its contrast is reduced in the next presentation of a letter. Likewise, if they fail to correctly identify the letter, the contrast is increased. This is repeated until thresholds are determined in 5 identical repeated trials. This normally involved around 70–100 separate presentations in total. Subjects were given an initial trial before testing to avoid a learning effect. Initial presentations were at high colour contrast. No learning effects were noted in the study.

## Results

### Light assessment in the experimental environment

**Fig. 1:**
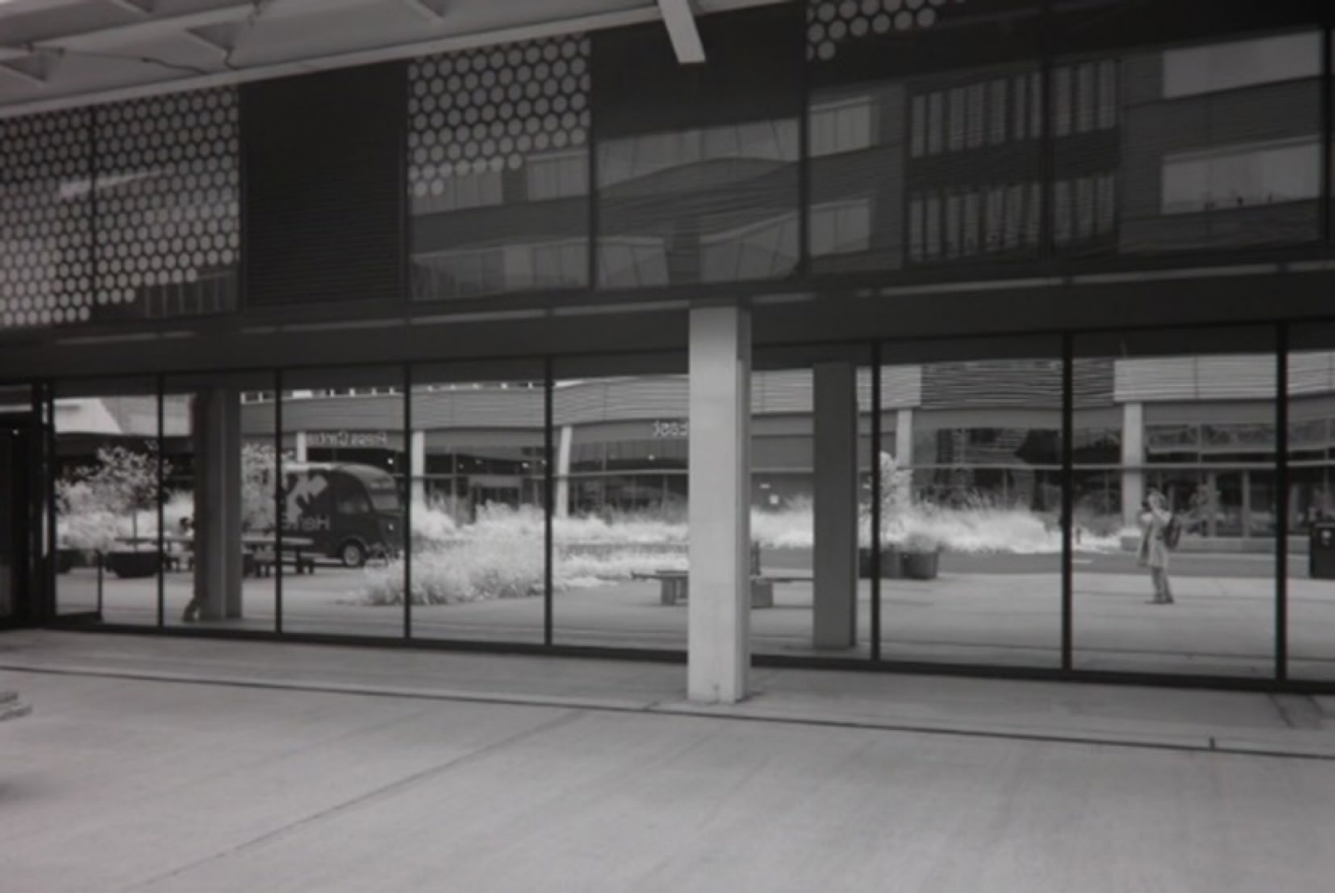
Infrared photograph of the front of the Here East building (~ 800–1000 nm). The glazing reflects infrared light away, resulting in the mirror-like appearance of the windows. The photographer (EB) can be seen on right hand side of the reflection.

**Fig. 2:**
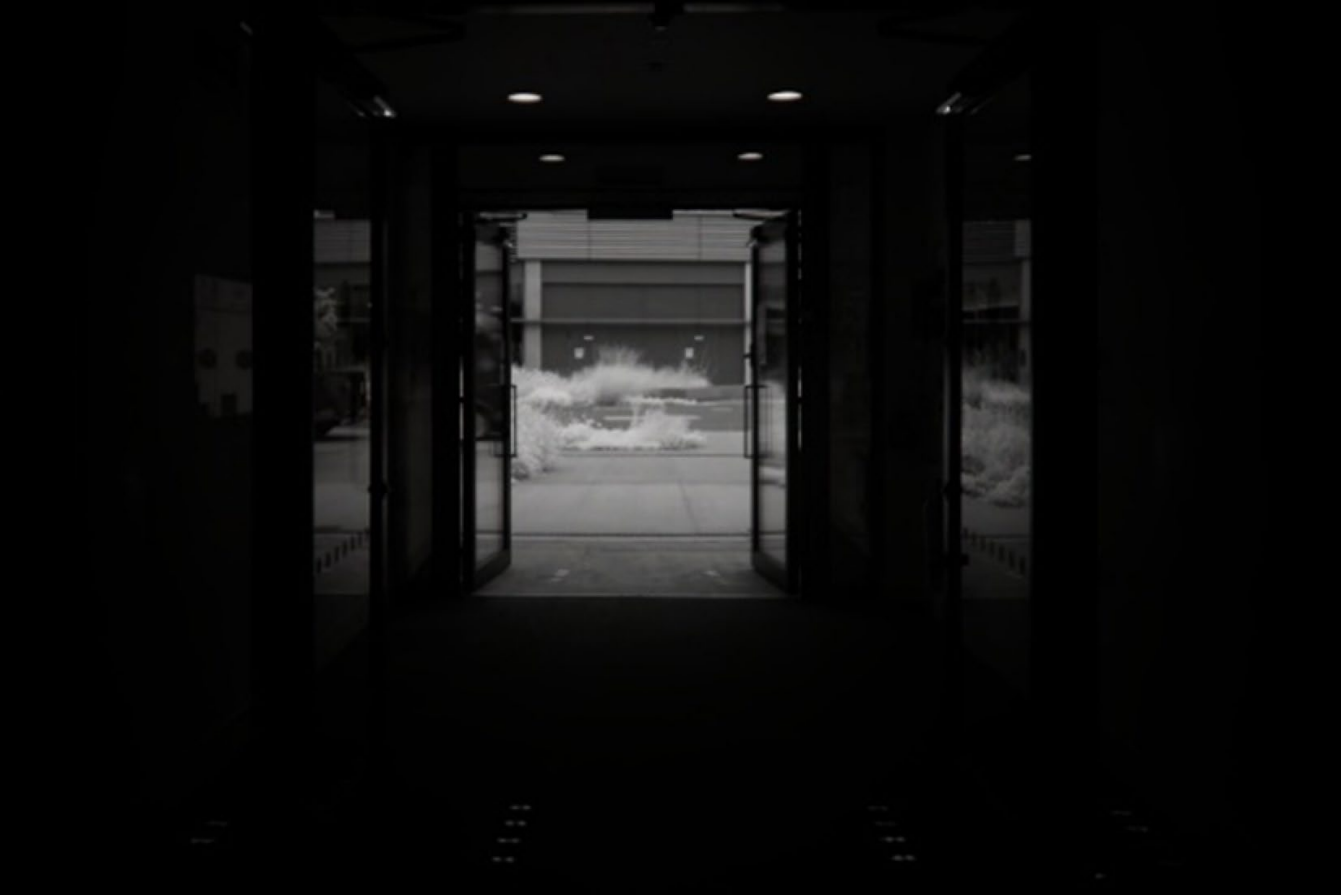
Infrared photograph (~ 800–1000 nm) taken from inside the Here East building, showing infrared light only entering the building when the front doors are opened. The interior space appears otherwise completely dark as infrared light is not passed by the glazing.

Figure 1 shows the front exterior of the Here East building using infrared imaging at ground level where experiments were undertaken. The windows are completely infrared reflective due to their blocking film and hence mirror-like. Figure 2 is an infrared image from inside the building looking out through the open doorway. Only infrared light coming through the open door and its reflectance can be seen, not light coming through adjacent windows. Hence, the building is relatively impervious to infrared light.

**Fig. 3:**
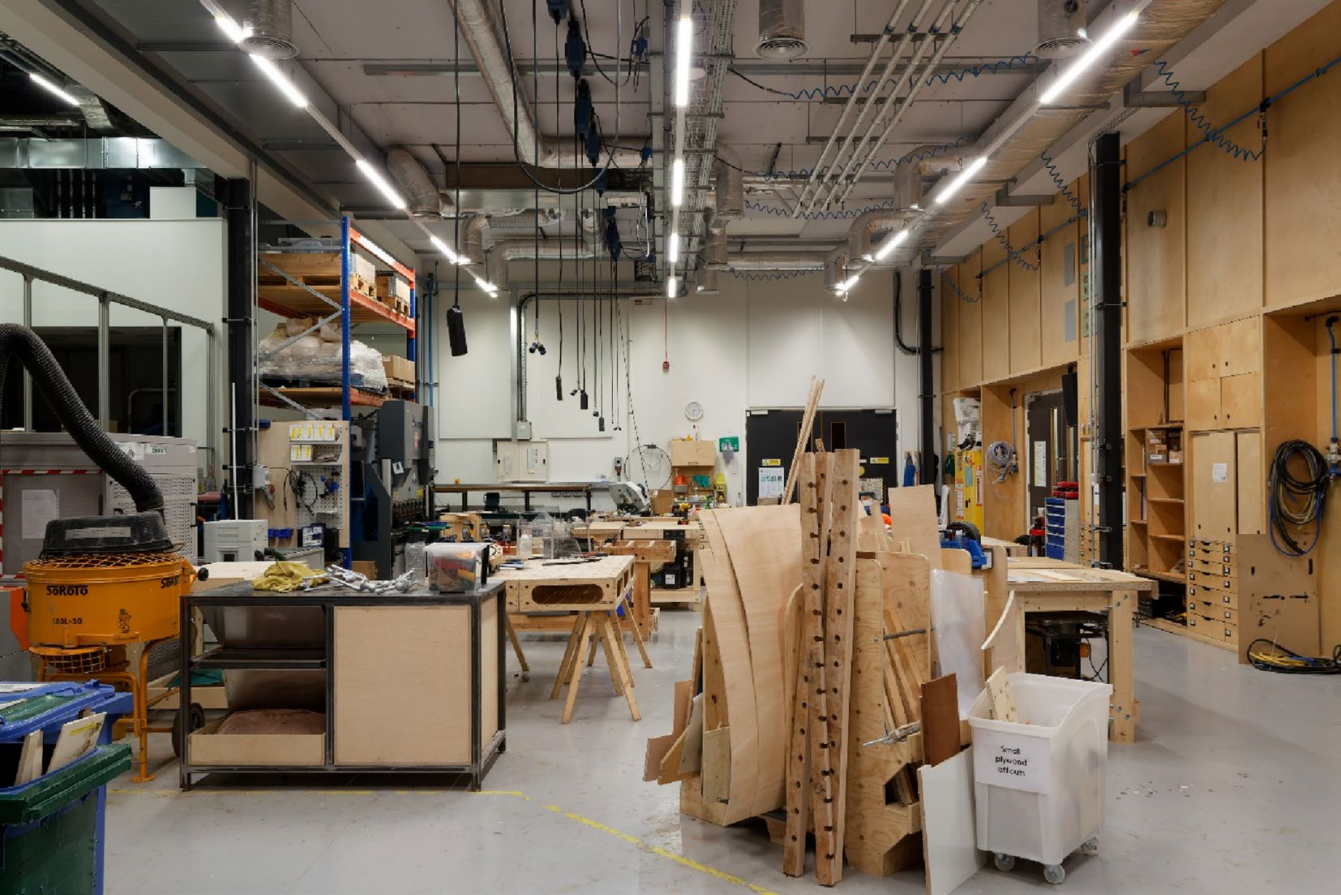
The work environment in Here East in which the LED lighting was supplemented with incandescent units. There was no natural light, and all lighting was in the form of overhead LEDs that can be seen in the ceiling mounts. The space was 9 m wide and 18 m deep. The linear array of LEDs were suspended at a hight of 4.25 m above the floor. The working hight of the benching is 0.9 m above the floor.

Figure 3 shows the working environment in Here East in visible light in which the experiment took place. Images in infrared were completely black. The distance between the work environment and the front door was > 50 m with multiple doors between.

**Fig. 4:**
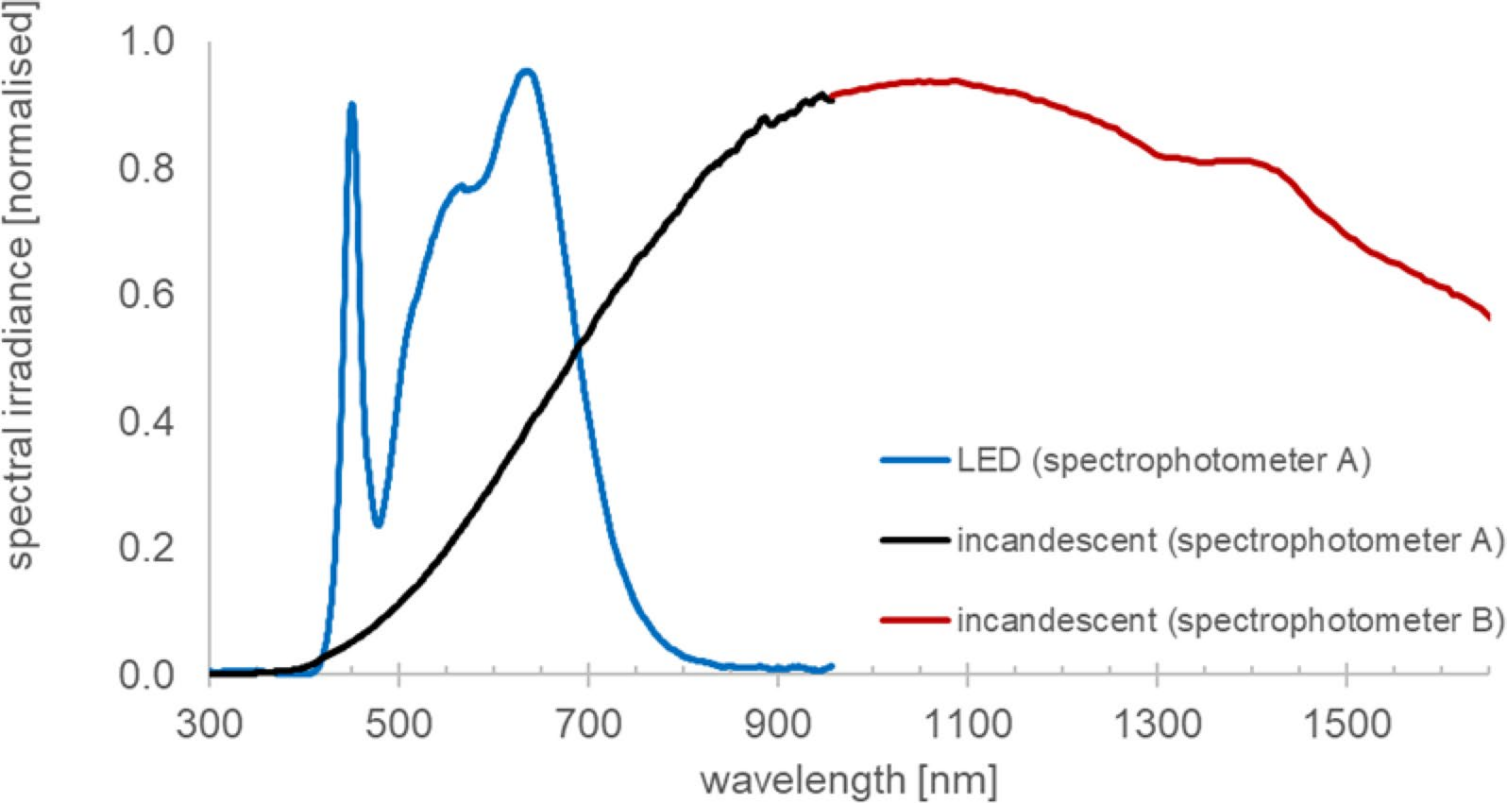
Spectral profiles of lighting. The light generated by the LEDs in Here East is shown in blue and runs from approximately 350 nm to 720 nm, with a sharp peak at 450 nm and a secondary shoulder between 500 nm and approximately 700 nm. The second spectra which is black and red is generated by a standard 60 W incandescent desk lamp. This is similar to sunlight extending from approximately 350 nm to > 1700 nm. The spectral profile of the incandescent is long wavelength shifted compared to the LED that lacks an extended infra-red component. The spectrum of this incandescent source extends beyond 2000 nm. However, the spectrometers used to measure this lacked sensitivity at longer wavelengths. Individual spectrometers have limits to the spectral range they can cover, so two have been used here and their different wavelength ranges are represented by the back and the red parts of the curve.

The internal lighting throughout Here East was provided by arrays of ceiling mounted standard LED units. Spectral profiles of the lighting within the building are shown in Fig. 4. against incandescent lighting in black and red.

The blue curve shows the spectral profile of the light delivered to the horizontal working plane. As the work environment was deep in the building and lit only with LED lighting, it received no daylight and was devoid of any infrared illumination. The LED units delivered 1000 lx on the horizontal working plane with a correlated colour temperature (CCT) of 4000 K and a TM-30 colour fidelity index of 91. The irradiance of this LED light was 3.6 W/m^2^.

**Fig. 5:**
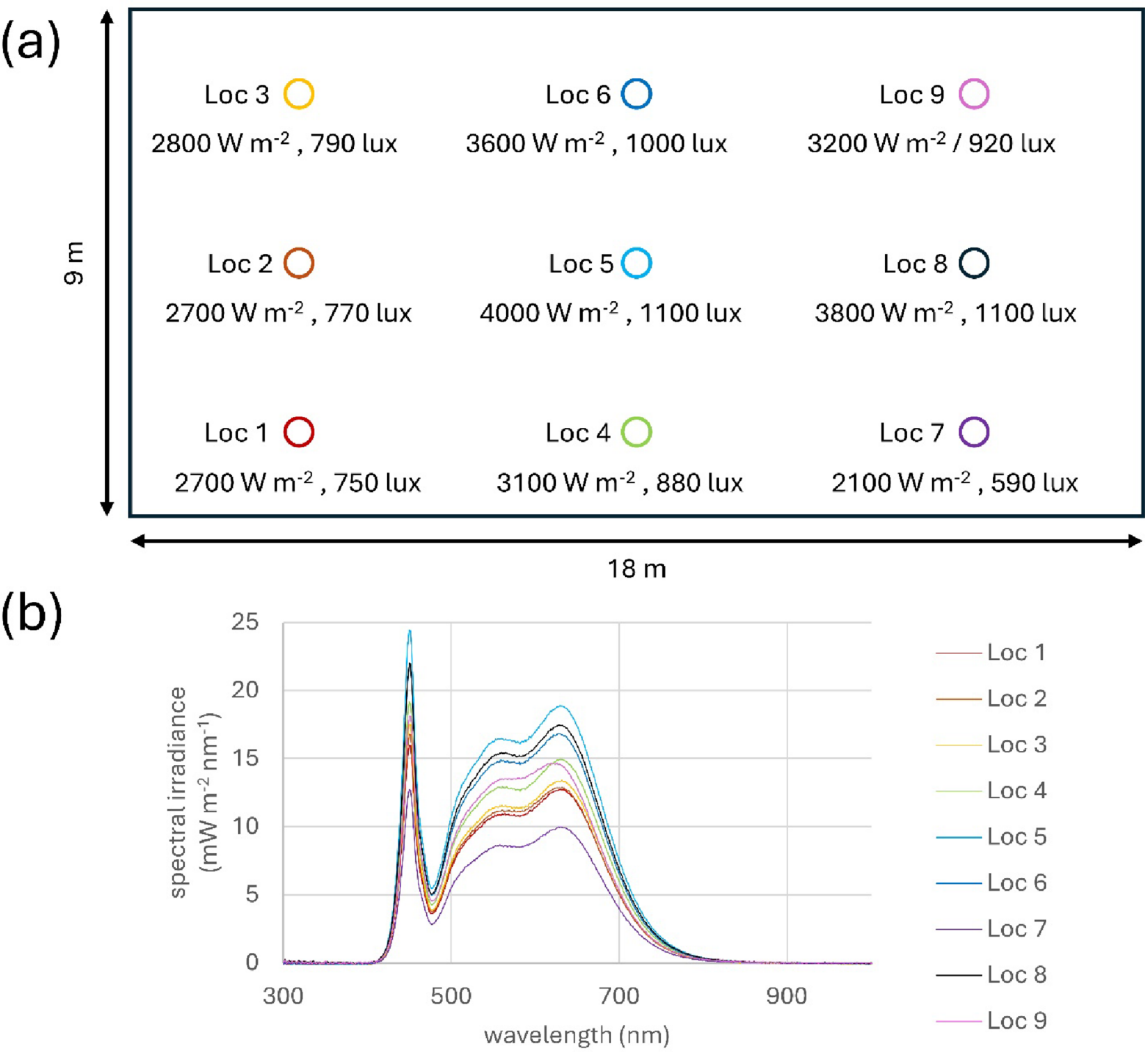
**a** The workshop area was 9 m wide and 18 m deep, with a ceiling at a height of 5 m. Horizontal illuminance was measured at nine locations (Loc 1 - Loc 9) on a grid of points evenly spaced through the workshop. Working height was 0.9 m above the floor. Typically, when working at their laptops the workers were near Loc 8, shown in black. (b) Individual spectral irradiance was also taken at the same nine locations, showing that there was the same spectral distribution provided throughout the space.

The specific spectra and energy levels were mapped over the workspace at fixed locations. This is shown in Fig. 5a and b. Here there is a plan of the space and measurements made at 9 locations of energy given in W/cm^− 2^ and lux, which is a metric corrected for the human eye. Also provided are the spectra at each location. Changes in brightness at different locations were largely not detectable by the human eye and were gradual. There were no differences in spectral profiles across the area only their relative intensity.

**Fig. 6:**
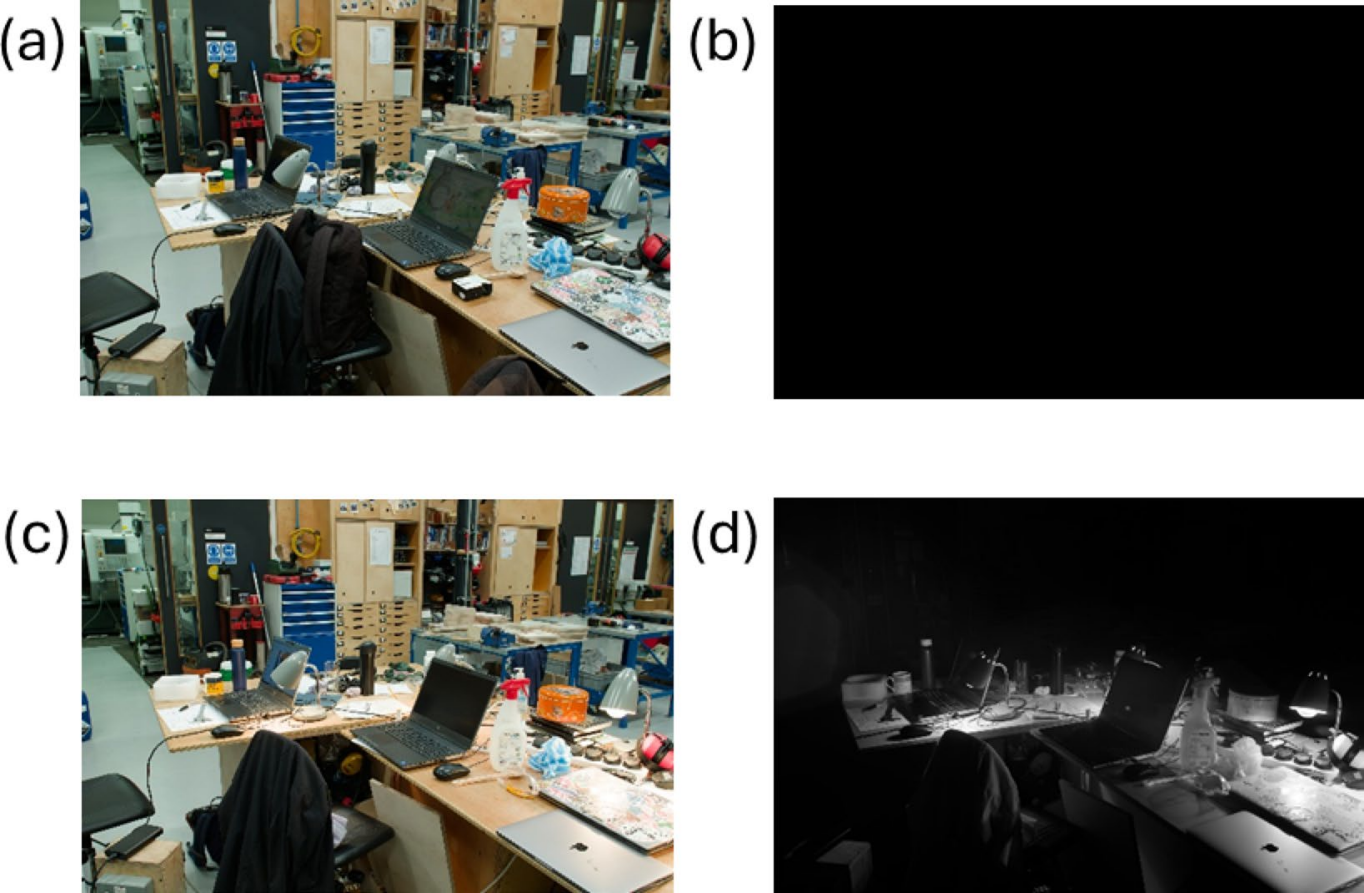
A series of photographs of the workshop area using the infrared sensitive camera with different filters. Photograph (**a**) shows the environment in visible light with the incandescent lighting off, and photograph (**b**) has the same with incandescent off but with infrared imaging. Photograph (**c**) shows the environment with the incandescent lighting on, and (**d**) shows the same with incandescent on and infrared imaging. Optical filters were used to isolate the visible and infrared parts of the spectrum respectively. Visible light was captured by placing an Astronomik “L-filter” which passes 390 nm to 670 nm, in front of the sensor. Infrared light was captured using an Astronomik “ProPlanet 807 IR” which passes 810 nm upwards, past the upper limit of 1000 nm for the sensitivity of the camera.

Light assessment was also undertaken at bench level where individual subjects worked. This is shown in Fig. 6. This confirmed the absence of any part of the infrared spectrum in the work environment and how this changed with the addition of the incandescent lamps.

**Fig. 7:**
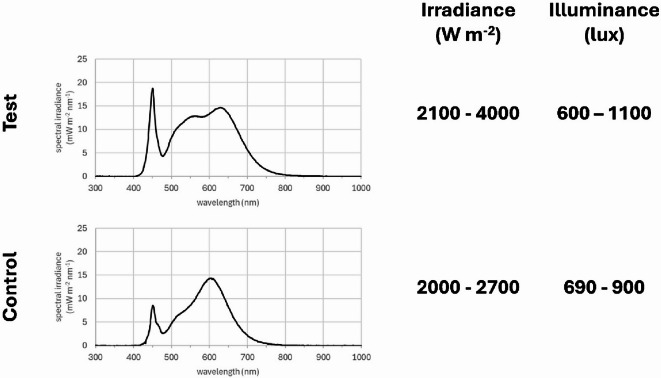
Examples of the spectra of LED lighting in the test and control areas. These are largely similar as were the LED specifications. Minor differences could be due to different reflective of the environments. The right side give both the luminance and the irradiance measurements of the two spaces. These overlapped and were very similar to the human eye. In both environments subjects were free to move around resulting in dynamic exposure to the lighting.

In this study, responses to lighting were measured in test subjects both before and also after the lighting had been changed. However, there was also an independent control group that comprised individuals under similar LED lighting condition without daylight. A comparison of the lighting conditions in the two groups is shown in Fig. 7. Critically, the LEDs in both groups had very similar profiles with no infrared components. The overall brightness in the control group was slightly less than in the test group, although this was not apparent to the human eye. As in the test group, subjects were free to move around.

### Visual responses to shifts in spectral lighting

**Fig. 8:**
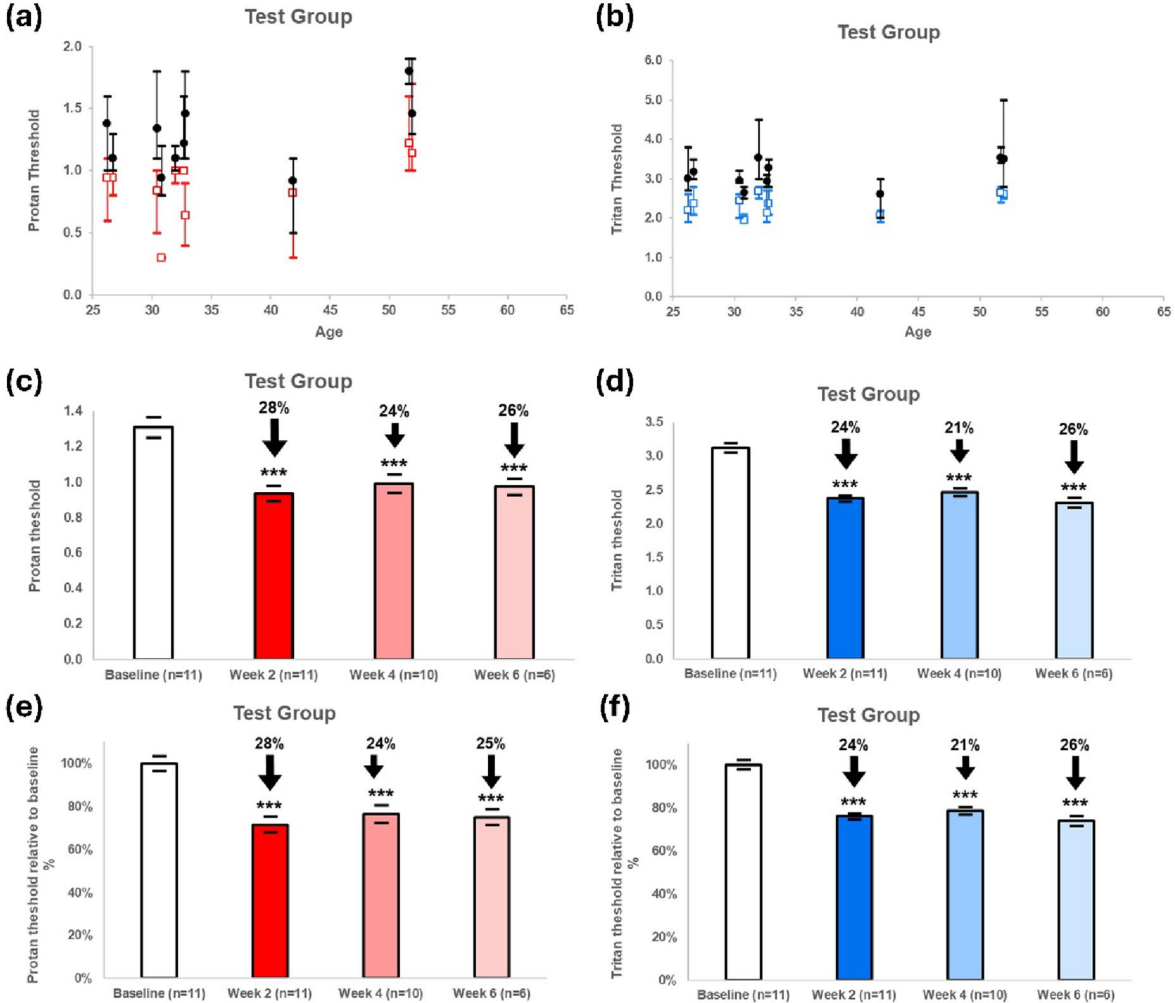
Protan (**a**) and tritan (**b**) thresholds measurements following 2 weeks incandescent light, showing data from individual subjects where the black circles are for baseline and the red squares are data following incandescent exposure for 2 weeks. In all cases the red post exposure data points are below the black demonstrating improved contrast. Below this is shown show the population change in threshold contrast sensitivity for protan (**c**) and tritan (**d**) at 2, 4, and 6 weeks after the incandescent lighting was removed. Arrows and percentages show the decline in thresholds across subjects. The degree of reduction was consistently similar for protan and tritan unlike exposures using 670 nm where improvements are biased to triton and only lasted 5 days. This indicates that the wider spectrum of incandescent lighting is having a greater impact on improving vision and lasting longer than previously experienced. The same result is seen if we look at data from individuals for the percentage change of this threshold across each subject (i.e. normalising for the baseline) for both proton (e) and tritan (f). Statistical symbols: *** p less than 0.001. Error bars standard error of the mean.

Exposure to 60 W incandescent luminaires, which have a wider spectrum than LEDs extending into the infra-red^1^, resulted in significant improvements in visual performance in all experimental subjects across both the protan and tritan visual ranges. Improvements in both tritan and protan were of the order of 25%. Hence, significant improvements were uniform across visual ranges (Fig. 8). This is unlike experiments where specific red/infrared ranges have been used in LED devices, for example via 670 nm, where visual improvements have been biased towards tritan function^13^.

Figure 8 shows the results of both individual subjects on the left and also changes in the groups on the right. In spite of the universal improvement in visual function, in both tritan and protan range there was considerable variability between subjects. This variability validates the inclusion of a repeated measures design and the use of a sign test in the analysis. In all cases protan thresholds were lower than tritan consistent with previous studies^13^.

At the end of the 2 week period the incandescent luminaires were removed and the subjects returned to an exclusively LED dominated light working environment. They were then retested at 4 and 6 weeks. In previous experiments where 670 nm alone has been used, rather than the wide spectrum infrared produced by incandescent lighting, visual improvements decline in approximately a week^13^. However, following incandescent light exposure improvements remain unchanged across both visual domains at both 4 and 6 weeks. Hence, the impact of broad-spectrum incandescent light not only resulted in balanced improvements in colour contrast but also these improvements lasted much longer than previous interventions with restricted red/infrared ranges^13^.

**Fig. 9:**
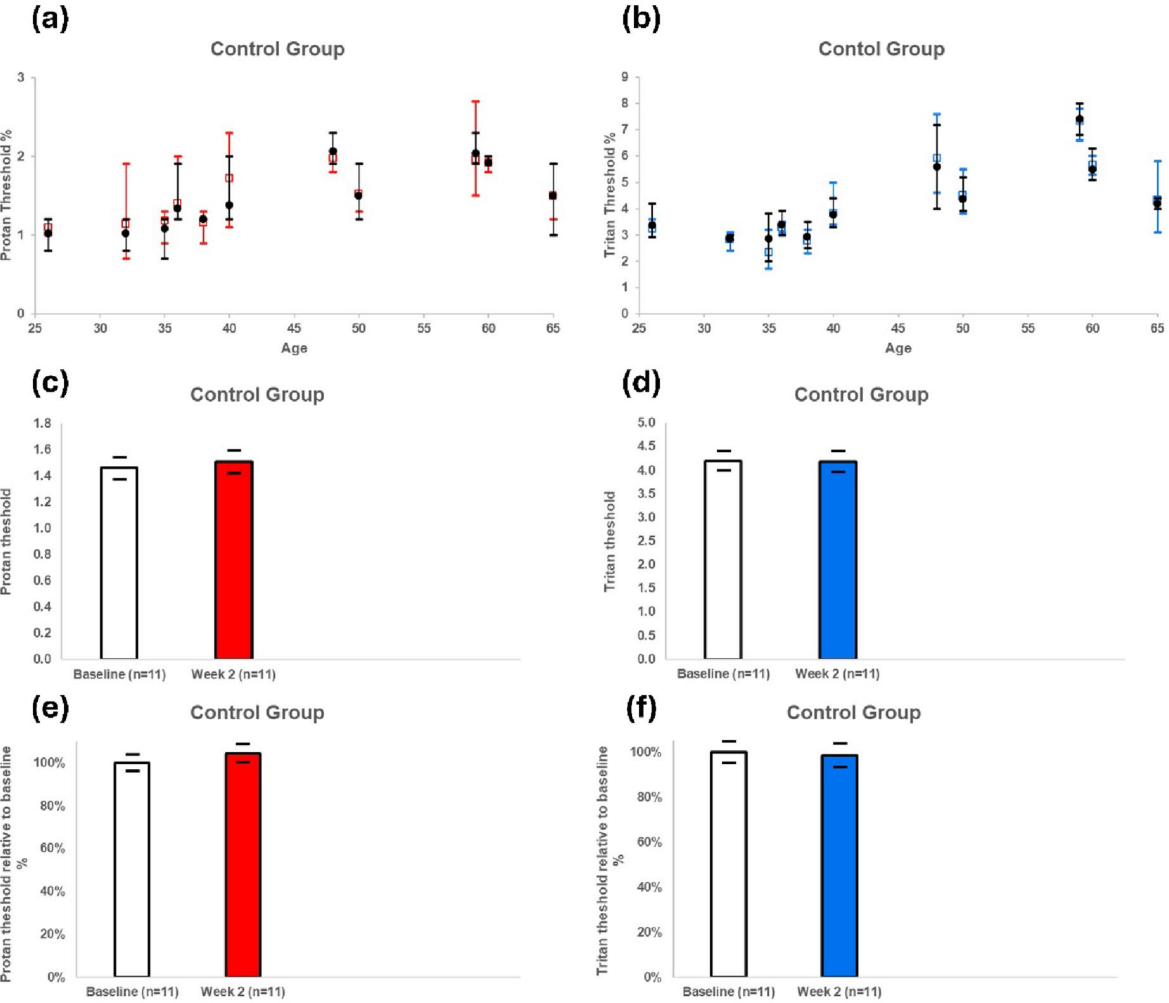
Proton (**a**) and tritan (**b**) threshold measurements as controls for those shown in Fig. 8. This shows data from individual subjects where the black circles are for baseline, and the coloured squares are data after two weeks under control conditions with no incandescent light added. Below this is shown the population threshold contrast sensitivity for protan (c) and tritan (d), which show no significant change over the two week period. As with Fig. 9, the same result is seen if we look individually at the percentage change of this threshold across each subject (i.e. normalising for the baseline) for both proton (e) and tritan (f). Abbreviations ns = Not significant. Error bars standard error of the mean.

An independent control group was used in addition to a before and after experimental design. Again, data between individuals was varied on both visual metrics. However, over a 2 week period there were no significant changes in proton or tritan visual thresholds (Fig. 9).

## Discussion

We demonstrate that the visual performance of those working under standard LED is significantly improved by exposure to incandescent lighting that has a spectrum similar to daylight with an extensive infrared component. These data are consistent with the hypothesis that LED lighting undermines human visual performance. This result is consistent with laboratory experiments where specific red/infrared wavelength ranges generated by LEDs have been used to improve visual function in animals and humans in a conserved manner^13,16,17^. But there are three critical differences from these earlier studies. First, we have simply changed environmental lighting in a free moving work environment. Second, we have obtained significant balanced improvements in both the protan and tritan range. Previously, exposure to restricted experimental 670 nm resulted in improvements biased strongly in favour of only tritan function^13^. Hence, exposure to full spectrum lighting results in a balanced pattern of improvement in visual performance. Third, we have shown that improvements in visual function following incandescent light exposure are sustained for up to 6 weeks, and possibly beyond, whereas benefits from single LED restricted range red light were confined to around 5 days^13^. These three features change the way in which long wavelength light may be applied to improve human physiology by delivery in normal environments with lasting balance effects. These results are novel and may have public health implications.

Our study used 22 subjects but was statistically significant using both a before and after metric and also against an independent control group. They are also similar to group sizes in aspects of Shinhmar et al.^13^ (Figs. 2, 3, 4 and 5). However, future studies would clearly benefit from inclusion of a larger number of subjects.

The evolution of life on earth extends over 4 billion years, and that of humans over approximately 4–5 million years from the last common primate ancestor. This has all taken place under sunlight that has a spectral range of approximately 300–2500 nm+, within which there has been an invariant balance between short and longer wavelengths. Human adoption of fire 1–2 million years ago supplemented sunlight as they moved out of Africa as its spectrum is similar having a large infrared component. Likewise, development of the Edison filament luminaire, common until approximately the year 2000 had a spectrum similar to sunlight. However, around 2010 LED lighting with its highly restricted spectrum (350–650 nm) and energy saving characteristics became common, resulting in a loss of infrared light in the built environment^1^.

The physiology of life forms are adapted to natural environmental light in a highly conserved pattern across species. Light impacts on mitochondrial function, which is a key regulator of metabolism and ageing in animals. When the balance of short and long wavelengths is shifted there are consequences for mitochondria. When shorter wavelength exposure is dominant, as in LED lighting, mitochondrial function declines. Mitochondrial complex proteins are reduced and there is reduced ATP production^2,3^. With reduced mitochondrial demand for glucose there is increased body weight and disruptions to serum cytokines^4^. Consequently, consistent with the mitochondrial theory of ageing there is an increased probability of cell/organism ageing and death^18^. It is suggested that this is partly due to 420–450 nm light, dominant in LEDs, being absorbed by porphyrin and the subsequent production of oxygen singlets driving inflammation^3^.

Conversely, exposure to longer wavelengths is associated with increased mitochondrial membrane potential and increased concentration of mitochondrial complex proteins that have declined with ageing and disease. This in turn is associated with elevated ATP, reduced inflammation and extended average lifespan^7,9,10,19^. The experimental use of longer wavelengths in such situations is commonly referred to as photobiomodulation.

The retina has the greatest metabolic rate in the body and a high mitochondrial concentration^20^. Retinal metabolism declines with age, but this can be partly corrected with long wavelength light across species^16,21^. In humans a single 3 min 670 nm exposure improves colour vision within 3 h, which is sustained for almost a week^13^. But what the authors of this study did not appreciate was that this was within a population who worked and lived mainly under LED lighting that may have undermined their baseline measurements. Here, we made no attempt to control light exposures or subject movements as would occur in laboratory-based experiments. Rather, our aim was to introduce wide spectrum long wavelengths into a work environment to improving human performance via mitochondrial manipulation in a translational step.

There is considerable evidence that introduction of longer wavelengths impact systemically. Durieux et al.^22^ stated in relation to experiments in C.elegans that “ We find that mitochondrial perturbation in one tissue is perceived and acted upon by the mitochondrial stress response pathway in distal tissue”. In mice there are significant distinct changes serum cytokine expression to exposures of both short and long wavelength light^4,23^. Similarly, long wavelength exposures to the surface of the human body excluding the eyes significantly reduces blood glucose levels and increases oxygen consumption in humans. This is likely because mitochondrial upregulation will increase carbohydrate demand to support increased ATP production^12^. Other systemic impacts can be found and are clear in experimentally induced Parkinson’s in primates. Light targeted by implants focusing on the substantia nigra are effective in reducing symptoms^24^, but so also are those that are directed at distal locations^25^.

Single 3 min 670 nm exposures remain effective for about 5 days^13^. But we show that with a wider spectrum they remain effective for 6 weeks, although we did not find the end of the effect. Here it is worth considering potential mechanisms of action which remain subject of debate. Historically, improvements with red light were thought to be due to light absorption by cytochrome C in the respiratory chain^26^. However, positive effects are found in vitro in the absence of this. Consequently, it has been suggested that longer wavelengths reduce water viscosity around rotary ATP pumps allowing the rotor to increase speed^27^. This cannot explain the sustained impacts of light exposure as this effect should be relatively transitory as viscosity would increase rapidly following light withdrawal. However, a key feature of long wavelength light absorption is increased respiratory chain protein synthesis. These proteins are in flux throughout the day^28^ and complex IV is upregulated following red light exposure^19^. Hence, while red light may initially increase rotor pump speed there rapidly follows an increase in protein synthesis which may establish greater respiratory chain capacity. The life of these proteins could then determine the length of effect.

Only thirteen polypeptides are made in mitochondrial protein synthesis. This probably slows with age and likely contributes to aged mitochondrial decline^18^. But critically, we do not know the speed of mitochondrial protein synthesis, the life of such proteins or the pace of their decline. We suggest that these may be key events in the length of the effects from light exposure.

LED lighting clearly has the ability to undermine visual performance probably via reduced mitochondrial function. As light induced changes in mitochondrial ability have been shown to have systemic impacts^4,15,22,23,25^, the effects of LEDs revealed here may be wider than initially anticipated. Given the prevalence of LEDs, this may represent an important issue in public health and clinical environments where changing lighting patterns in appreciation of this point can have significant positive outcomes^29^.

Given our results, it is important to ask what solutions may be found to improve health in terms of lighting in the built environment. Incandescent lights that we reveal here to have significant positive impact over standard LEDs are being phased out universally for reasons of energy efficiency, where focus is only on the visible light produced.

A solution may be found in creating lighting units with multiple longer wavelength LEDs to cover a wider span of the near infrared. However, our attempts here have had limited success. Multiple closely associated spectral peaks do not produce a smooth spectral output as found in incandescent lights and sunlight, which is problematic in improving function and has yet to deliver. This possibly may be overcome using a greater number of spectral peaks with tighter spacing. But this raises a different series of problems regarding cost and increased energy consumption making this solution no better than retention of incandescent sources in terms of environmental sustainability.

Key to this issue is the question of how much infrared is needed to sustain improved function? Infrared has relatively few absorbers in the built environment and in current studies relatively little has to be added to the environment for effect. However, a viable option is to run an incandescent light at a lower temperature which results in both energy savings and increased life of the unit and also shifts the peak spectral output towards longer wavelengths.

If this is done with a halogen bulb, which is a type of incandescent tungsten bulb, the filament lasts for a longer period as evaporated tungsten is redeposited on the filament rather than blackening the bulb glass. Hence, using a halogen bulb at lower voltage is a realistic alternative in terms of health and energy consumption.

## Data Availability

The data sets used and/or analysed during the current study are available from the corresponding author on reasonable request.
